# Cytokine-induced killer (CIK) cell therapy for patients with hepatocellular carcinoma: efficacy and safety

**DOI:** 10.1186/2162-3619-1-11

**Published:** 2012-04-26

**Authors:** Yue Ma, Ying-Chun Xu, Lei Tang, Zan Zhang, Jian Wang, Hong-Xia Wang

**Affiliations:** 1 Department of Oncology, Shanghai Renji Hospital, Shanghai Jiaotong University School of Medicine, Shanghai 200127, China; 2 Department of Surgery, Shanghai Renji Hospital, Shanghai Jiaotong University School of Medicine, Shanghai 200127, China

**Keywords:** Cytokine-induced killer cells, Hepatocellular carcinoma, Clinical trial, Meta-analysis, Therapy

## Abstract

**Purpose:**

To evaluate the efficacy of cytokine-induced killer (CIK) cell therapy in the treatment of hepatocellular carcinoma.

**Materials and methods:**

Randomized phase II and III trials on CIK cell-based therapy were identified by electronic searches using a combination of "hepatocellular carcinoma" and "cytokine-induced killer cells".

**Results:**

The analysis showed significant survival benefit (one-year survival, *p *< 0.001; two-year survival, *p *< 0.001; median overall survival, *p *< 0.001) in favor of CIK-based therapy. Comparison of CIK group versus non-CIK group resulted in a significantly prolonged progression-free survival (PFS) (*p *< 0.01). A favored disease control rate (DCR) and overall response rate (ORR) were also observed in patients receiving CIK cell therapy (*p *< 0.01). Meanwhile, patients in the CIK group showed better quality of life (QoL), diminished HBV-DNA content and AFP level (*p *< 0.01). Comparing T-lymphocyte subsets in peripheral blood, the analysis showed the ratio of CD3^+^, CD4^+^, CD4^+^CD8^+ ^and CD3^+^CD4^+ ^T cells significantly increased in the CIK group, compared with the non-CIK group (*p *< 0.01).

**Conclusions:**

CIK cell therapy demonstrated a significant superiority in prolonging the median overall survival, PFS, DCR, ORR and QoL of HCC patients. These results support further larger scale randomized controlled trials for HCC patients with or without the combination of other therapeutic methods.

## Introduction

Hepatocellular carcinoma (HCC) is the third most common cancer globally, with a poor prognosis and limited systemic treatment options [1]. In men, it is the fifth most common cancer worldwide and the third-leading cause of cancer-related death [2]. HCC is resistant to conventional chemotherapy and is insensitive to radiotherapy. Surgery, transcatheter arterial chemoembolization (TACE) and radiofrequency ablation (RFA) are considered as the main treatments for HCC today [3]. However, the recurrence rate is still high, and long-term survival is unsatisfactory, as approximately 80% of patients die within a year of diagnosis. After curative resection or transplantation, tumor recurrence rate can be as high as 25% per year. Although some centers have reported excellent long-term results, survival after hepatic resection or transplantation is as low as 50% at 3 years and 20%-30% at 5 years [4]. Therefore, finding effective methods to strengthen treatment efficacy and prevent recurrence is an important issue in HCC therapy.

Cytokine-induced killer (CIK) cells, which are non-major histocompatibility complex (MHC)-restricted CD3^+^CD56^+ ^T cells, take advantage of the body's natural ability to eliminate tumor cells by stimulating and restoring the immune system to recognize and kill tumor cells [5]. Majority of CIK cells express T cell receptors, and others express NK cell markers. CIK cells are generated by incubating mononuclear cells from peripheral blood, bone marrow or cord blood with various types of additions. Current protocols to differentiate CIK cells are based on a combination of interferon (IFN)-γ on day 1 of culture, followed by CD3 monoclonal antibody (CD3McAb), interleukin2 (IL2), interleukin1 (ILl) 24 hours later [6,7]. CIK cells have higher proliferation rate, cytolytic activities and non-MHC-restricted killing of tumor cells in comparison with lymphokine-activated killer cells (LAK cells) which are essentially activated by natural killer (NK) cells [8,9].

Clinical studies indicated that autologous CIK cell therapy could be used as an efficient adjuvant anticancer immunotherapy to eradicate residual cancer cells, prevent recurrence, improve progression-free survival (PFS) rates, and promote the quality of life (QoL) for cancer patients [10-14]. Therefore, we performed a systematic review and meta-analysis of randomized controlled clinical trials (RCTs) to assess the efficacy and tolerability of CIK cells in the treatment of patients with HCC.

## Materials and methods

### Study design, search strategy, and eligibility criteria

Trials were identified by electronic searches in the PubMed database, the Cochrane Central Registry of Controlled Trials, the Wanfang Database, the China Science and Technology Periodical Database, China Journal Net, reference lists of published trials and relevant review articles. The search strategy included the medical subject headings of "hepatocellular carcinoma", "cytokine-induced killer cells" and free text searches. No language limits were applied. Initial searches were performed in August 2011, with updates in February 2012. In addition, we contacted drug manufacturers, asked experts in the field, and performed manual searches in reference lists, conference proceedings of the American Society of Clinical Oncology (ASCO) Annual Meetings and the European Cancer Conference (ECCO). We excluded abstracts that were never subsequently published as full papers and studies on animals.

### Data collection

We gathered information including authors' names, journal and year of publication, sample size per arm, performance status (PS score), regimen used, median age of patients, and information pertaining to study design (whether the trial reported the mode of randomization, allocation concealment, description of withdrawals per arm, and blinding) for the trials included in the study. Written informed consent was obtained from the patient for publication of this report and any accompanying images.

### Definition of outcome measures

Overall survival (OS) and the PFS were the primary outcome measure. OS was defined as the time from the initiation of treatment until death from any cause. PFS was defined as the time from the initiation of treatment to the first observation of disease progression or death from any cause. The secondary endpoints were the overall response rate (ORR) and disease control rate (DCR). Toxicity was graded according to the NCI Common Toxicity Criteria. QoL was assessed by the Karnofsky performance status (KPS) [15].

### Statistical analysis

The analysis was performed using a Review Manager Version 5.0 (Nordic Cochran Centre, Copenhagen). We defined a statistical test with a p value less than 0.05 as significant. Odds ratio (OR) and 95% confidence interval (CI) as relevant effect measures were estimated directly or indirectly from the given data. Where they were not provided, they were estimated indirectly from other summary statistics or from the data extracted from published Kaplan-Meier curves. To assess statistical heterogeneity among trials, the Cochran's chi-square test (*Q *test) was performed, with a predefined significance threshold of 0.05. If the *Q *test was statistical significant (*p *< 0.05), a random effects meta-analysis was performed; otherwise, a fixed effect model was used. All reported p values result from two-sided versions of the respective tests. The revision of funnel plots did not reveal any considerable publication bias.

## Results

### Selection of the trials

**Figure 1:**
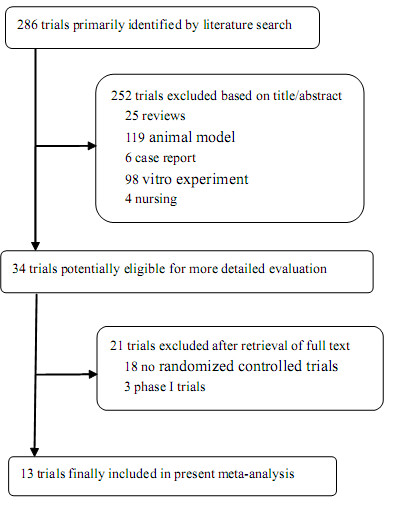
**Flow diagram of the study selection process**.

The electronic searches yielded two hundred eighty six references. After title and abstract review, 252 publications were excluded for different reasons (25 for being review articles, 119 for using animal models, 6 for being case reports, 98 for being vitro experiments, 4 for being nursing studies). The full texts of 34 articles were selected as potentially relevant and retrieved for more detailed assessment. We excluded a total of 21 studies for the following reasons: 3 trials were excluded for being phase I clinical trials, 18 trials were excluded for being non-RCTs. The selection procedure of the clinical trials is shown in Figure 1. As a result, 13 articles reporting phase II and III clinical trials of CIK cell-based therapy were selected for meta-analysis. These 13 eligible RCTs included a total of 1212 patients.

### Characteristics of CIK cell-based therapy

Clinical data of these trials are listed in Table 1. CIK therapy combined with TACE in four of the trials [16-19], with TACE and RFA in other four of the trials [20-23], with surgery alone in three trials [24-26], with TACE alone or TACE and percutaneous ethanol injection (PEI) in other two trials [27,28] were evaluated. IFN-γ, CD3McAb, IL-1a and IL-2 were used in CIK cell culture system in all of the analyzed trials.

**Table 1:** Clinical information of the eligible trials for the meta-analysis

Trial	No. of pts	Regimens (per arm)	No. of pts (Male)	CIK Regimens	Culture of CIK cell
Dong 2009 [24]	127	CIK (3 course)	41 (31)	1.0-2.0 × 10^10 ^per course	CM, IFN-γ, CD3McAb, IL-1a, IL-2

		CIK (6 course)	43 (32)		

		Surgery only	43 (34)		

Weng 2008 [20]	85	TACE + RFA + CIK	45 (31)	1.0-1.5 × 10^10 ^per course	CM, IFN-γ, CD3McAb, IL-1a, IL-2

		TACE + RFA	40 (29)		

Zhao 2006 [21]	64	TACE + RFA	31 (29)	1.1-1.5 × 10^10 ^per course	CM, IFN-γ, CD3McAb, IL-1a, IL-2

		TACE + RFA + CIK	33 (30)		

Pan 2010 [22]	83	TACE + RFA + CIK	42 (37)	> 1.0 × 10^10 ^per course	CM, IFN-γ, CD3McAb, IL-1a, IL-2

		TACE + RFA	41 (34)		

Hao 2010 [19]	146	TACE	74 (64)	1.0-5.0 × 10^10 ^per course	SFM, IFN-γ, CD3McAb, IL-1a, IL-2

		TACE + CIK	72 (65)		

Lu 2008 [25]	30	CIK	12 (UK)	1.6 × 10^10 ^per course	SFM, IFN-γ, CD3McAb, IL-1a, IL-2

		Surgery alone	18 (UK)		

Zhang 2007 [16]	44	TACE	20 (UK)	8.0 × 10^9 ^per course	CM, IFN-γ, CD3McAb, IL-1a, IL-2

		TACE + CIK	24(UK)		

Guo 2007 [17]	61	TACE	31 (UK)	1.0-1.2 × 10^10 ^per course	CM, IFN-γ, CD3McAb, IL-1a, IL-2

		TACE + CIK	30 (UK)		

Zhang 2006 [27]	144	TACE	30 (UK)	1.0-1.2 × 10^10 ^per course	CM, IFN-γ, CD3McAb, IL-1a IL-2

		TACE + CIK	16 (UK)		

		TACE + PEI	62 (UK)		

		TACE + PEI + CIK	36 (UK)		

Shi 2007 [28]	252	TACE	134 (UK)	1.0-1.2 × 10^10 ^per course	CM, IFN-γ, CD3McAb, IL-1a, IL-2

		TACE + PEI	80 (UK)		

		TACE + CIK	38 (UK)		

Hao 2006 [18]	67	TACE + CIK	21 (17)	1.0-5.0 × 10^10 ^per course	CM, IFN-γ, CD3McAb, IL-1a, IL-2

		TACE	46 (45)		

Wan 2008 [23]	61	TACE + RFA	34 (23)	1.0 × 10^10 ^per course	CM, IFN-γ, CD3McAb, IL-1a, IL-2

		TACE + RFA + CIK	27 (18)		

Yu 2009 [26]	50	TACE + CIK	25 (22)	1.0-1.2 × 10^10 ^per course	CM, IFN-γ, CD3McAb, IL-1a, IL-2

		Surgery alone	25 (23)		

Note: *UK *unknown; *pts *patients; *TACE *transcatheter arterial chemoembolization; *RFA *radiofrequency ablation; *PEI *percutaneous ethanol injection; *IL *interleukin; *IFN-γ *Interferon-γ; *CM *complete medium; *SFM *Serum-free culture medium

The CIK cells for all trials were prepared from peripheral blood. The number of CIK cells transfused into patients in these studies ranged from 8.0 × 10^9 ^to 5.0 × 10^10 ^per course. The patient information from two groups (CIK cell therapy and non-CIK cell therapy) of the trials such as gender and CIK cell dose were analysed by *χ*^2 ^test. There was no statistically significant difference between groups (p > 0.05). Different article-origin of the patient information in each group did not interfere with the results of meta-analysis.

### Survival

**Figure 2:**
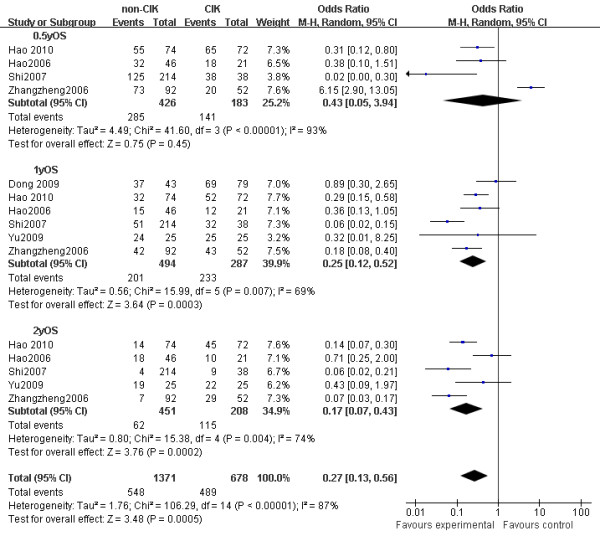
**Comparison of 0.5-year, 1-year, and 2-year survival between non-CIK group and CIK group**. OR, odds ratio; OS, overall survival. non-CIK, non-CIK-containing therapy; CIK, CIK-containing therapy. The random effects meta-analysis model (Mantel-Haenszel method) was used in this analysis. Each trial is represented by a square, the center of which gives the odds ratio for that trial. The size of the square is proportional to the information in that trial. The ends of the horizontal bars denote a 95% CI. The black diamond gives the overall odds ratio for the combined results of all trials. The center denotes the odds ratio, and the extremities denote the 95% CI.

The analysis showed that significant survival benefit (OS: OR = -20.01, 95% CI: -25.72 to -15.31, *p *< 0.001) was observed in patients receiving CIK-based therapy. The results of the pooled analysis showed that CIK arm was associated with significantly improved one-year survival (OR = 0.25, 95% CI: 0.12 to 0.52, *p *< 0.001) and two-year survival (OR = 0.17, 95% CI: 0.07 to 0.43, *p *< 0.001). However, there was no difference in half-year survival comparing the CIK group versus non-CIK group (77% in CIK group versus 67% in the non-CIK group; OR = 0.43, 95% CI: 0.05 to 3.94, *p *= 0.45) (Figure 2).

**Figure 3:**
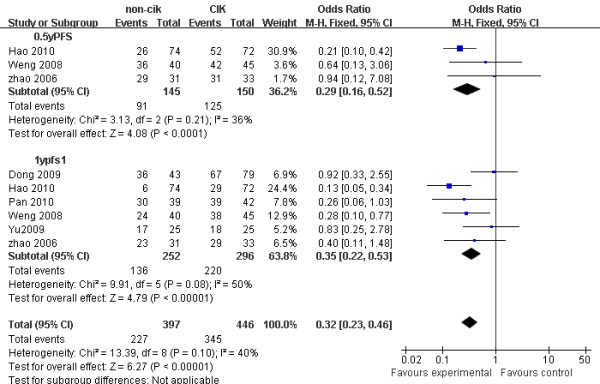
**Comparison of 0.5-year, 1-year PFS between non-CIK group and CIK group**. OR, odds ratio; PFS, progression-free survival; non-CIK, non-CIK-based therapy; CIK, CIK-based therapy. The fixed effects model (Mantel-Haenszel method) was used in this analysis.

Concerning PFS, treatment with CIK-combined therapy was also associated with a significantly prolonged half-year PFS (OR = 0.29, 95% CI: 0.16 to 0.52, *p *< 0.001) and one-year PFS (OR = 0.35, 95% CI: 0.22 to 0.53, *p *< 0.001) (Figure 3).

### Response rate

**Figure 4:**
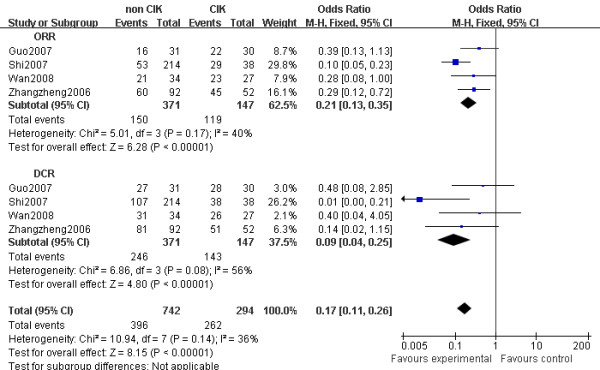
**Forest plot for ORR and DCR**. non-CIK, non-CIK-based therapy; CIK, CIK-based therapy; DCR, disease control rate; ORR, overall response rate. The fixed effects meta-analysis model (Mantel-Haenszel method) was used in the analysis.

The analysis of DCR and ORR also demonstrated favorable results for the CIK cell therapy arm (OR = 0.09, 95% CI: 0.04 to 0.25, *p *< 0.001 and OR = 0.21, 95% CI: 0.13 to 0.35, *p *< 0.001) (Figure 4).

In the subgroup analysis, a significantly prolonged DCR (OR = 0.08, 95% CI: 0.02 to 0.40, *p *= 0.002) and ORR (OR = 0.36, 95% CI: 0.17-0.72, *p *= 0.004) were observed in the patients treated with CIK combined TACE therapy compared with those treated with TACE combined PEI therapy.

### Toxicity, HBV-DNA content and plasma AFP

In most trials, slight fever and chills could be seen, and the body temperature varied from 37.5°C to 39.0°C within 24 hours after CIK cell transfusion. The overall OR of 0.07 (95% CI: 0.01 to 0.53) demonstrated that the incidence of fever in the CIK therapy group was significantly higher than those in the non-CIK group (*p *= 0.01).

We classified QoL as "improvement", "stability" or "deterioration", if KPS was higher than, equal to or lower than pretreatment, respectively. The analysis showed that TACE combined CIK therapy can improve HCC patients' QoL, showing a better QoL (OR = 0.32, 95% CI: 0.16 to 0.64, *p *= 0.001) when compared with non-CIK therapy. The HBV-DNA content in the analysis was based on two HCC trials [21,22]. During one-year follow-up, no patient HBV-DNA content was more than 1 × 10^6 ^copy/ml in the CIK therapy group. Patients in the CIK group had lower HBV-DNA content than patients in the non-CIK group (OR = 27.5, 95% CI: 5.21 to 145.15, *p *< 0.01). (Table 2).

**Table 2:** Fever, improvement of QoL and HBV-DNA in trials included in the analysis

Event	No. of pts (%)		OR	95% CI	p value
				
	Non - CIK	CIK			
Fever	0	12.40%	0.07	0.01 - 0.53	0.010
QOL improvement	58.54%	76.83%	0.32	0.16 - 0.64	0.001
QOL stability	31.70%	19.51%	2.52	1.22 - 5.20	0.010
QOL deterioration	9.76%	3.66%	2.91	0.79 - 10.64	0.110
HBV-DNA (> 1 × 10^3 ^copy/ml)	91.30%	27.59%	27.50	5.21 - 145.15	< 0.010

Summary differences of fever, improvement of QoL and HBV-DNA were calculated using the random-effects model.

Note: *QoL *quality of life; *HBV-DNA *Hepatitis B virus DNA; *pts *patients; *CIK *CIK-containing therapy; *OR *odds ratio; *CI *confidence interval.

**Figure 5:**
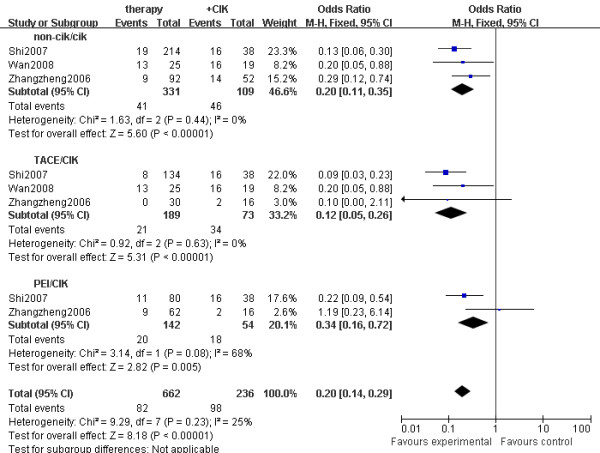
**Forest plot for AFP concentration drop to normal in different therapy group**. non-CIK, non-CIK-based therapy; CIK, CIK-based therapy; TACE, transcatheter arterial chemoembolization therapy; PEI, percutaneous ethanol injection therapy. The fixed effects meta-analysis model (Mantel-Haenszel method) was used in this analysis.

The AFP content in the analysis was based on three HCC trials ^23,26.27^. Plasma AFP decreased more significantly in the CIK group than in the non-CIK group (OR = 0.20, 95% CI: 0.14 to 0.29, *p *< 0.001). Plasma AFP of patients in the CIK group was more likely to drop to a normal level, compared with the non-CIK group (OR = 0.20, 95% CI: 0.11 to 0.35, *p *< 0.001), the TACE alone group (OR = 0.12, 95% CI: 0.05 to 0.26, *p *< 0.001) and the PEI group (OR = 0.34, 95% CI: 0.16 to 0.72, *p *= 0.005) (Figure 5).

### Comparison of T-lymphocyte subsets in peripheral blood

The analysis showed the ratio of CD3^+^, CD4^+^, CD4^+^CD8^+ ^and CD3^+^CD4^+ ^T cells significantly increased in the CIK group, compared with the non-CIK group, which was reflected by a pooled OR of -0.79 for CD3^+ ^cells (95% CI:-1.13 to -0.45, *p *< 0.001),-2.00 for CD4^+ ^cells (95% CI:-2.7 to -1.3, *p *< 0.001), 0.04 for CD4^+^CD8^+ ^cells (95% CI: 0.03 to 0.05, *p *< 0.001), and -2.02 for CD3^+^CD4^+ ^cells (95% CI:-2.27 to -1.76, *p *< 0.001). Furthermore, the percentage of CD8^+ ^and CD3^+^CD8^+ ^T cells significantly decreased in the CIK group compared with the non-CIK group (95% CI: 2.43 to 3.67, *p *< 0.001; 95% CI:-2.1 to -1.56, *p *< 0.001; respectively) (Table 3).

**Table 3:** Immunophenotype assessment in different therapy group

T cell	No. of trials	No. of patients		MD	95% CI	p value
					
		Non-CIK	CIK			
CD3^+^	6	276	162	-0.79	-1.13 to -0.45	< 0.001
CD4^+^	5	258	150	-2.00	-2.70 to -1.3	< 0.001
CD8^+^	5	258	150	3.05	2.43 to 3.67	< 0.001
CD3^+ ^CD8^+^	2	57	54	-1.83	-2.10 to -1.56	< 0.001
CD4^+ ^CD8^+^	6	285	188	0.04	0.03 to 0.05	< 0.001
CD3^+ ^CD4^+^	2	57	54	-2.02	-2.27 to -1.76	< 0.001

Summary differences of immunophenotype assessment in different therapy group were calculated using the fix-effects model. To assess statistical heterogeneity between studies, the Cochran Q test was performed, with a predefined significance threshold of 0.1.

Note: *CIK *CIK-containing therapy; *CI *confidence interval; *MD *mean difference

## Discussion

According to research in recent years, HCC patients have some immune dysfunctions, including those in innate and adaptive immune responses [29]. It has been reported that interferon therapy appeared to decrease recurrence rate after resection of hepatitis C virus or hepatitis B virus-related HCC in some randomized controlled trials. Tumor immunological studies show that cellular immunity of cancer patients is closely related to the occurrence and development of cancers. Cytokine immunotherapy not only has fewer side effects but also can avoid tumor dysimmunity and specific tolerance of tumor antigen. With the rapid advance of molecular biology technology, the application of immunotherapy combined with surgery or interventional therapy is thought to be promising strategy of HCC treatment.

Schmidt-Wolf et al. [5] first reported that CIK cells had a strong anti-proliferative capacity and cytotoxicity on tumor cells. Further studies have demonstrated that CIK cells, which are lymphocytes induced by many cytokines [30], have better anti-tumor effects compared with LAK cells (lymphocytes activated by IL-2 alone). Our analysis showed that CIK cell therapy was associated with significantly prolonged one-year and two-year survival, OS and PFS, but had no effect on half-year survival (*p *= 0.45). A favored DCR and ORR were also observed in patients receiving CIK cell therapy (*p *< 0.01). The mechanism of anti-tumor activity of CIK cells is still unclear. Schmidt-Wolf et al. [31] demonstrated that perforin-mediated pathways possibly play an important role in CIK cells induced tumor cell killing effect.

The study also indicated that patients receiving CIK cell therapy had improved QoL compared with patients in the non-CIK group (*p *< 0.01). Although CIK group was associated with more fevers (*p *= 0.01). However, fever after CIK cell transfusion was light in most trials and lasted only 24 hours or less. In biological treatments, moderate fever is considered to be a normal reaction of immune function and beneficial to treatment [32].

We also observed that the HBV-DNA and AFP levels decreased significantly in the CIK group (*p *< 0.01). Hepatitis B virus infection can lead to hepatic sclerosis and HCC. AFP is currently widely recognized as a tumor-related prognosis antigen for HCC. HCC patients with high levels of HBV-DNA and AFP have a poor prognosis [33,34]. Concomitant infection with HBV and impairment from hepatic sclerosis in the hepatic parenchyma lead to an increase of AFP levels [35,36]. The reduction of AFP content and HBV-DNA content contribute to preventing the short-term recurrence of HCC and prolonging patients' survival time.

Targeting of the human immune system against tumor mainly depends on cellular immunity. CD4^+ ^T cells are considered to have a predefined role as a helper T cell within the immune system, providing help in recruiting CD8^+ ^T cells and activating macrophages through IFN-γ production. It has been demonstrated that cytotoxicity against tumor is dependent on an appropriate CD4^+ ^and CD8^+ ^T cell interaction. The ratios of T lymphocyte subsets in peripheral blood are usually disordered in tumor patients [37,38]. The analysis showed the percentage of CD3^+^, CD4^+ ^and CD3^+^CD4^+ ^T cells significantly increased in the CIK group, compared with the non-CIK group, but the percentage of CD8^+ ^and CD3^+^CD8^+ ^T cells significantly decreased in the CIK group, compared with non-CIK group (*p *< 0.001). The percentage of CD4^+ ^T cells significantly increased, CD8^+ ^T cells significantly decreased, and thus the ratio of CD4^+^/CD8^+ ^increased. Therefore, immune suppression was attenuated, enhancing the immune system's tumor clearance ability.

In present study, CIK cells were cultured in complete medium (CM) supplemented with human blood serum in eleven trials, and other two trials used serum-free medium (SFM) to culture CIK cells. Mitomycin, cisplatin, anthracycline and lipiodol were used for TACE. Most studies showed that the culture of CIK cells amplified more and produced more IFN-γ, IL-4, or IL-5 in CM than in SFM [39,40]. Our analysis showed that half-year PFS, one-year PFS, one-year survival in the CM were 93.6%, 85.3%, 84.2% which differ significantly from the 72.2%, 40.3%, 72.2% in CIK cells cultured in the SFM. However, half-year survival (68.5%) and two-year survival in the CM (51.5%) were lower than those in SFM group (90.3%, 72.2%).

The present meta-analysis was not based on individual patient data and was not subjected to an open external evaluation procedure. Therefore, the analysis is limited in that the use of published data may have led to an over-estimation of the treatment effects. With respect to both response and survival, we could not limit our analysis to intention-to-treat populations as the total number of patients randomized per arm was not always reported. Therefore, for consistency among studies, we elected to use the assessable patients for our analysis. Moreover, all the selected trials in present study were conducted in Asia, lacking multinational larger sample multicenter clinic research with sufficient statistical power. In order to solve this problem, a larger scale international multicenter randomized clinical trial should be conducted in the near future.

Taken together, the CIK cells were prepared after in vitro priming and were transfused into patients with HCC. These early results appear very promising, and the side effects related to CIK cell transfusion were few. It will hopefully lead to more large and controlled clinical trials in these settings.

## Conclusion

CIK cell therapy demonstrated a significant superiority in prolonging the OS, PFS, DCR, ORR and QoL of HCC patients compared with non-CIK therapy. These observations support further larger scale RCTs to evaluate the efficacy of CIK cell therapy in the treatment of HCC with or without the combination of other therapeutic methods.

## Competing interests

The authors declare that they have no competing interests.

## Authors' contributions

LT and YCX performed the computerized search of the trials, contacted experts and participated in the trial selection. YM and ZZ participated in the trial selection and performed the statistical analysis. HXW and JW conceived of the study. All authors read and approved the final manuscript.
